# Virginia

**Published:** 1896-10

**Authors:** 


*Virginia.* The Board of Health of Norfolk received in private sessions a report from Dr. Faville as to the inspection of the various dairy-herds in the vicinity of that city, all supplying milk to its citizens, and we note in connection with this an advertisement inserted in the papers of Norfolk by those milk-producers who have had their herds tested and who have eliminated all the tuberculous animals. Some of them have adopted monthly inspection, so as to insure clean and healthy milk to their customers. One of such herds had some nine animals removed from it as tuberculous.

Veterinarian Harbaugh, of Richmond, very thoroughly discussed, in the July *Bulletin* of the Virginia Board of Health, the subject of bovine tuberculosis as it relates to State medicine. Entering upon the history of tuberculosis from the time of the discovery of tubercular bacilli, he took up the subject of the transmission of tuberculosis from one species of animal to another by association and through feeding-experiments and the exhalations of diseased human subjects to other lower animals. He forcibly calls attention to the danger of leaving tuberculous animals in dairy-herds and to the danger of milk from such sources, and the necessity of the State taking up, through its central and local boards of health, the question of protecting the people from the dangers lying in animal food-products offered for sale on the public markets, without the consumers having any information as to the condition of the animals from a health point of view or to the surroundings and conditions under which these important foods were produced and preserved. Touching upon the point as to the frequency of diseases in thoroughbred or high-bred cattle, he showed that it was not a question of high- or low-bred cattle, but as to its propagation among herds through the introduction of a single tuberculous animal. In closing his appeal, he quotes the following from Nocard's work on animal tuberculosis: "It seems not unreasonable to suppose that the same is the cause for human tuberculosis, and that, if the children of tuberculous parents were protected from infection by cohabitation or ingestion, the importance of heredity as a cause of the disease, or even of the predisposition to it, would dwindle away into insignificance."

A vigorous and unrelenting war should be made against animal tuberculosis as one of the great causes of human tuberculosis, and this war should be a campaign of extermination, as well as of education.

